# A meta-analysis on heart rate variability biofeedback and depressive symptoms

**DOI:** 10.1038/s41598-021-86149-7

**Published:** 2021-03-23

**Authors:** Silvia F. M. Pizzoli, Chiara Marzorati, Daniele Gatti, Dario Monzani, Ketti Mazzocco, Gabriella Pravettoni

**Affiliations:** 1 Department of Oncology and Hemato-Oncology, University of Milan, Via Festa del Perdono 7, 20122 Milan, Italy; 2 Applied Research Division for Cognitive and Psychological Science, European Institute of Oncology IEO, IRCCS, Milan, Italy; 3 Department of Brain and Behavioral Sciences, University of Pavia, Pavia, Italy

**Keywords:** Physiology, Psychology, Health care

## Abstract

Heart rate variability biofeedback (HRVB) has been used for a number of years to treat depressive symptoms, a common mental health issue, which is often comorbid with other psychopathological and medical conditions. The aim of the present meta-analysis is to test whether and to what extent HRVB is effective in reducing depressive symptoms in adult patients. We conducted a literature search on Pubmed, ProQuest, Ovid PsycInfo, and Embase up to October 2020, and identified 721 studies. Fourteen studies were included in the meta-analysis. Three meta-regressions were also performed to further test whether publication year, the questionnaire used to assess depressive symptoms, or the interval of time between T0 and T1 moderated the effect of HRVB. Overall, we analysed 14 RCTs with a total of 794 participants. The random effect analysis yielded a medium mean effect size *g* = 0.38 [95% *CI* = 0.16, 0.60; 95% *PI* =  − 0.19, 0.96], *z* = 3.44, *p* = 0.0006. The total heterogeneity was significant, *Q*_*T*_ = 23.49, *p* = 0.03, *I*^2^ = 45%, which suggested a moderate variance among the included studies. The year of publication (*χ*^2^(1) = 4.08, *p* = 0.04) and the questionnaire used to assess symptoms (*χ*^2^(4) = 12.65, *p* = 0.01) significantly moderated the effect of the interventions and reduced heterogeneity. Overall, results showed that HRVB improves depressive symptoms in several psychophysiological conditions in adult samples and should be considered as a valid technique to increase psychological well-being.

## Introduction

Depression is one of the most common mental health conditions, globally affecting about 265 million people of all ages (World Health Organization^1^). Low mood, loss of interest, sleep disturbances, reduced appetite, slowed thinking and lack of energy have been identified as potential risk factors for the onset and persistence of other diseases: people suffering from depression are more likely to have substance use issues, personality disorders, or other psychological problems (e.g., distress, anxiety)^2^.

A worldwide study conducted on 245,404 people from 60 different countries, showed that patients suffering from chronic diseases were more likely to show comorbidity with depression^3^. Moreover, depressive symptoms are often associated with poor health outcomes^4^, noncompliance with medications and treatments^5^, and are often comorbid with medical issues such as type 2 diabetes^6^ and dementia^7^.

Furthermore, depressive symptoms are associated with a higher risk of cardiovascular disease^8,9^. Patients with heart disease tend to show a higher prevalence of comorbid depression, while post-stroke survivors frequently report depressive symptoms up to 5 years after the cerebrovascular event^10–12^.

Several interventions have been developed to alleviate depressive symptoms: psychotherapy, antidepressant medication, and physical exercises being the most frequent treatments employed^13–15^. Beyond pharmacological treatment, the Health Evidence Network^16^ reported that the most effective psychotherapeutic interventions for the depression management are supportive counselling and psychodynamic, interpersonal or cognitive behavioural therapies (CBT). Specifically, different studies have shown that drugs and CBT are equally effective at reducing depressive symptoms in acute, short-term conditions^17^; while CBT has demonstrated greater mid-term and long-term effects than antidepressants^14^. In recent years, new psychotherapeutic approaches have focused on treating depression: mindfulness-based cognitive therapy (MBCT)—a combination of CBT and mindfulness meditation practice—and the so-called “third-wave” therapies have reported significant improvements in depressive symptoms^18–20^. However, these psychotherapeutic approaches do not directly focus on the regulation of physiological outcomes that are highly affected in depressive syndromes^21–23^. Besides psychosocial factors, negative emotional symptoms of depression are associated with autonomic nervous system responses, thus involving skin conductance, respiratory and heart rates^21,24,25^.

A key marker of the autonomous nervous system function and a potent predictor of physical morbidity and mortality is heart rate variability (HRV), a measure of the variation in time between each heartbeat. Greater variability indicates greater ability of the autonomic nervous system to regulate itself. This parameter may be used as a diagnostic and predictive bio-marker of depression, since more severe symptoms are significantly associated with reduced HRV^26–28^ and reduced HRV itself seems to be implicated in the risk of developing depression^29^.

HRV findings led to the implementation of a new technique widely used in several physical illnesses and mental disorders: HRV Biofeedback (HRVB), a non-invasive therapy training aiming at increasing heart rate oscillations through real time feedback and slow breathing training^30^. This intervention has been implemented for issues in regulating HRV, which were observed in depression treatment^31,32^. Previous studies demonstrated that HRVB improves HRV as measured by standard deviation of normal-to-normal intervals (SDNN), high-frequency power (HF) and low-frequency power/high-frequency power ratio (LF/HF). All of these physiological indices are associated with amelioration of depressive symptoms^26,32,33^.

Numerous studies have demonstrated the positive effect of HRVB in reducing physical and psychological symptoms and increasing wellbeing^34–38^. Furthermore, two meta-analyses were recently conducted to assess the efficacy of biofeedback on mental health.

Goessl et al. conducted a random-effects meta-analysis on the effects of HRVB on symptoms of anxiety and stress, finding that the HRVB is a useful and effective technique for improving self-reported stress and anxiety^39^.

Lehrer et al.^40^ recently performed a systematic and meta-analytic review on the efficacy of HRVB and/or paced breathing (approximately six breaths/min) on a wide range of psychological symptoms (including depressive symptoms), mental functions and complex behaviours (such as athletic/artistic performance). The investigators found a significant small effect of HRVB and paced breathing on depression^24^.

However, no meta-analyses have been specifically conducted on randomized controlled studies to investigate the specific effect of HRVB in adults with depressive symptoms (i.e. patients with depressive disorders or with depressive symptoms in comorbidity with other psychological or physical conditions). To fill this gap, the aim of our meta-analysis is to estimate the effect of HRVB in reducing depressive symptoms.

## Material and methods

### Search strategy and inclusion criteria

To identify potential studies for inclusion in the meta-analysis, we conducted a search of the published literature using the following scientific online databases: Pubmed (all years), Proquest (all years), Ovid PsycInfo (all years), and Embase (all years). Search criteria were: (“heart rate variability biofeedback” OR “HRV biofeedback”) AND (“depression” OR “depressive”). No time restrictions were applied. The full search strategies were reported in Online Appendix A.

The literature search was conducted up to October 2020.

### Study selection

One of the authors conducted a systematic literature search. Two other authors selected papers for full review based on inclusion and exclusion criteria and assessed their eligibility. Agreement was reached on the final selection of included studies.

Inclusion criteria were: (1) English-language publication, (2) work included an HRV biofeedback intervention, (3) randomized clinical trial (RCT), (4) peer-reviewed publication, and (5) work involved adult participants.

### Review strategy and data extraction

A total of 721 articles were identified and retrieved. After the first screening of title and abstract, 134 studies remained. After a full-text examination, 95 studies were excluded. 2 studies reported incomplete data^41,42^; corresponding authors were contacted, none provided the requested data, and those studies were therefore excluded. The remaining 14 studies were included in the present meta-analysis^26,35,43–54^.

**Figure 1:**
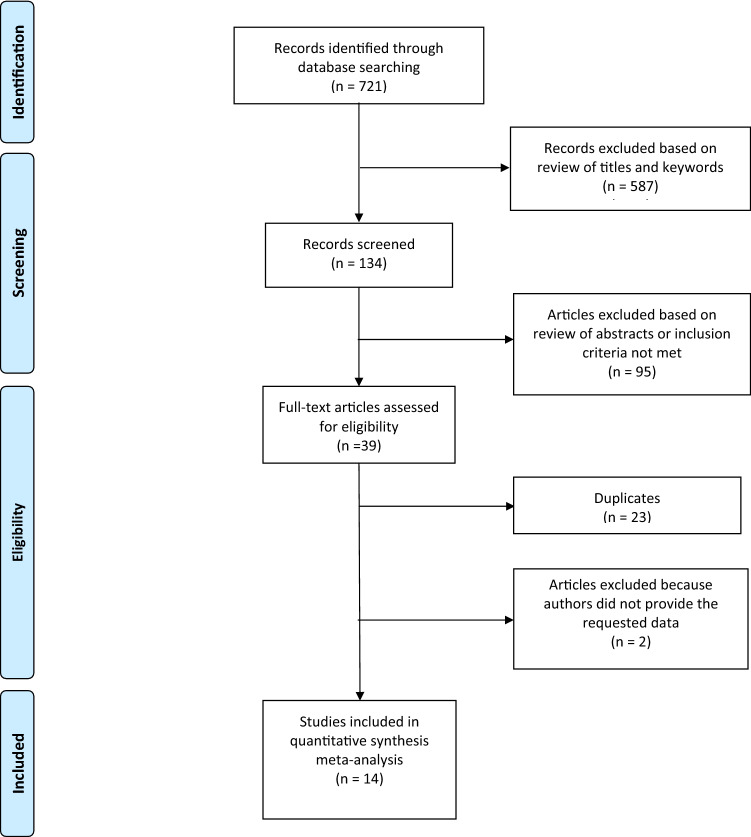
Flowchart illustrating study selection, review strategy and data extraction.

Preferred Reporting Items for Systematic Reviews and Meta-Analyses (PRISMA) guidelines were applied. The flowchart is shown in Fig. 1.

### Effect size calculation

The level of depressive symptoms was our dependent variable of interest. From each study we extracted: sample size, and mean and standard deviation of participants’ scores in the various conditions for the variable of interest.

The effect size used was *Hedges’ g*^55^, which is a standardized mean difference that accounts for sampling variance difference between conditions. The effect size and variance calculation were performed using R-Studio (RStudio Team 2015) and its package compute.es^56^ using the command *mes* when mean and standard deviations were available or *pes* when only *p*-values were reported. Effect sizes were computed comparing participants measures at time 1 (T1) between intervention vs. control group. This criterion was violated only for one study^45^, in which T1 was at 2 weeks and time 2 (T2) at 5 weeks; in this case we used T2 values. We decided to use the data collected at T2 in order to improve timing coordination with the other included studies, which all had T1s at least at 4 weeks after the end of the training.

### Risk of bias assessment

Risk of bias was assessed with the tool recommended by Cochrane guidelines^57^. Included RCTs were analysed according to random sequence generation, allocation concealment, blinding of participants and personnel, blinding of outcome assessment, incomplete outcome data, selective reporting, and other bias. Each source of bias was rated as yes (“low risk of bias”), no (“high risk of bias”), or unclear (“moderate risk of bias”). Disagreements in bias scoring were resolved by discussions among the two reviewers.

### Data analysis

In order to assess whether HRVB can successfully reduce depressive symptoms and quantify the effect of the modulation, we performed one random-effects meta-analysis using the restricted maximum-likelihood estimation method. We also carried out three distinct meta-regressions to assess whether the year of publication of the study, the test used to evaluate depressive symptoms, or the timing of T1, moderated the observed effect.

The within-studies heterogeneity was evaluated using the *Q*-test. A significant *p*-value of the *Q*-test implies that the observed within-studies variance can be explained by other variables besides HRVB. In addition, we used as index of heterogeneity Higgins’ *I*^2^^58^, which provides the percentage of the total variability in the effect size estimation that could be attributed to heterogeneity among the true effects (heterogeneity is considered high if *I*^2^ > 75%, Higgins et al.^58^). To further investigate heterogeneity, we also computed prediction intervals (*PI*) of the effect, which quantify the dispersion of effect. That is, 95% *PI* indicate the range of values that the effect size of a future study similar to those included should probably take (Borenstein et al.^55^).

Publication bias was evaluated using funnel plots and the trim-and-fill method (Duval 2005). The trim-and-fill method provides an estimate the number of studies missing from the meta-analysis due to the suppression of the most extreme results on one side (generally the left, i.e., non-significant results) of the plot. To further explore the publication bias, the Egger’s test^59^ was performed. The Egger’s test examines the correlation between the various effect sizes and their sampling variances (i.e., if the funnel plot is asymmetric), and a significant *p*-value indicates publication bias^60^. To explore the robustness of the results, we performed a leave-one-out analysis: this procedure evaluates the robustness of the effect excluding one study at a time. The meta-analyses performed, and the related plots were computed using the R-package metafor^61^.

## Results

### Summary of findings

The characteristics of the included trials are reported in Online Appendix B. The studies included in our meta-analysis were conducted between 2009 and 2020. The sample size varied from 20 to 134; all the studies used a between-subject design. A total of 14 RCTs were analysed on a total of 794 patients divided into experimental (*M* age = 46.17 *SD* = 7.72, 42.72% female) and control (*M* age = 46.81, *SD* = 7.17, 44.5% female) groups. Among the studies, two were conducted in Taiwan^35,43^, two in Netherlands^44,47^, three in the USA^26,46,54^, three in Germany^45,49^, one in the UK, one in Austria, one Iran and one in Sweden^48,50,51,53^. Nine studies were conducted on patients with cardiovascular disease, stress symptoms, cancer or alcohol disorder, while the remaining five studies focused on non-clinical samples^26,44,47,48,52^.

Of the 14 studies included, all evaluated depression with reliable instruments: six assessed depression through the BDI-II^62^, four through the DASS^63^, two through the HADS^43^, one employed the PHQ-9^64^ and one the CED-S^65^. Overall, the time range of the follow up ranged from five weeks post end-of-intervention^48,53^, to 12 months after the intervention^35,53^.

### Risk of bias

**Figure 2:**
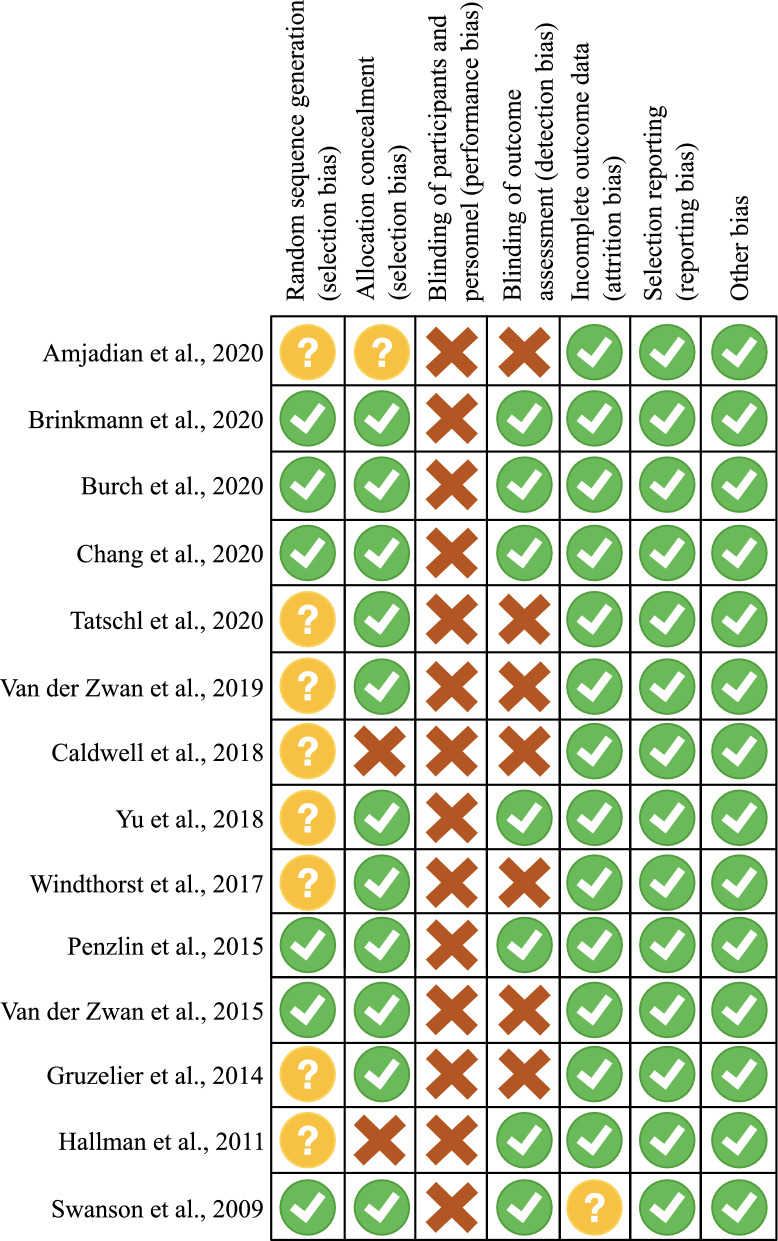
Risk of bias assessment. Low risk of bias is represented with green dots, high risk of bias in red, unclear risk of bias in yellow.

Figure 2 reported risk of bias assessment. None of the included studies withheld information on interventions from trial participants. Eight trials did not report how randomization was performed. In seven studies, the assessment of outcomes by researchers was blinded. Overall, most information was from trials at low or unclear risk of bias.

### HRVB and depressive symptoms

**Figure 3:**
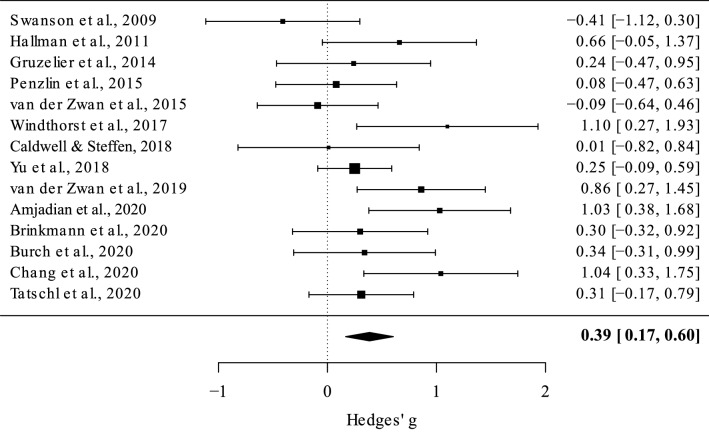
Forest plot, each square corresponds to one study and the lines represent 95% confidence interval. The size of each square represents the weight of the study. The diamond at the bottom represents the cumulative effect size with 95% confidence interval. Higher positive values indicate greater effect of the HRVB intervention compared with the control group.

The random effect analysis (*N* = 14) showed a medium mean effect size, *g* = 0.38 [95% *CI* 0.16, 0.60; 95% *PI* =  − 0.19, 0.96], *z* = 3.44, *p* = 0.0006, meaning that HRVB has a positive effect in reducing depressive symptoms. Total heterogeneity was significant, *Q*_*T*_ = 23.49, *p* = 0.03, *I*^2^ = 45%, suggesting moderate variance across the studies included (Fig. 3).

**Figure 4:**
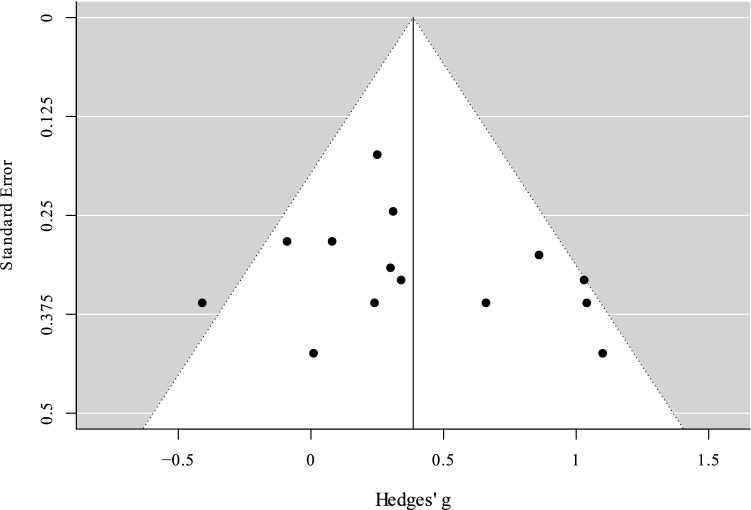
Funnel plot. Black dots represent studies included in the present meta-analysis. The vertical line represents the effect size.

The sensitivity analysis showed that the effect size ranged between 0.33 and 0.42 (*M* = 0.38, *SD* = 0.03). The trim-and-fill method added no hypothetical missing studies on the left side of the funnel plot (Fig. 4). The Eggers test was not significant, *z* = 0.85, *p* = 0.39, suggesting no publication bias.

**Figure 5:**
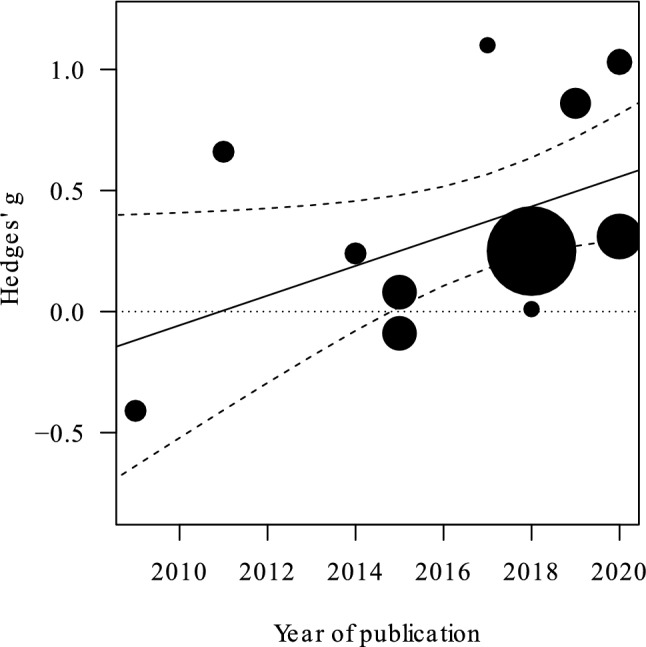
Plot illustrating the relationship between year of publication and cumulative effect sizes. In particular, the most recent the study, the greater the effect size. The area of the points is proportional to study variances. Three studies published in 2020 are not reported due to overlapping positions. Dashed lines indicate 95% CI, the dotted line indicate Hedges' g = 0 (i.e., no difference between groups).

The meta-regression performed using the year of publication as moderator (*N* = 14) showed that the test on the moderator was significant, *χ*^2^(1) = 4.08, *p* = 0.04, *estimate* = 0.06. The heterogeneity became not significant, *Q*_*T*_ = 18.14, *p* = 0.11, and Higgins’ *I*^2^ decreased, *I*^2^ = 0%. The decrease in heterogeneity suggests that the year of publication plays a role in determining the differences in the effects reported by the various studies; in particular, most recent studies reported higher effect sizes (Fig. 5).

The meta-regression performed using the test used to evaluate depressive symptoms as moderator (*N* = 14) showed that the test on the moderator was significant, *χ*^2^(4) = 12.65, *p* = 0.01. The heterogeneity became not significant, *Q*_*T*_ = 10.23, *p* = 0.33, and Higgins’ *I*^2^ decreased, *I*^2^ = 7%. The decrease in heterogeneity suggests that the test used to evaluate depressive symptoms plays a role in determining the differences in the effects reported by the various studies. Critically, when depressive symptoms were assessed using CES-D, the effect size was not significantly different from zero, while when they were measured by means of BDI-II, DASS, HADS or PHQ-9 the effect size was significantly different from zero (Table 1).

**Table 1:** *Hedges’ g* calculated using the questionnaire used to evaluate depressive symptoms as moderator.

TEST	BDI-II	CES-D	DASS	HADS	PHQ-9
*Hedges’ g*	0.23	− 0.41	0.48	0.85	1.10
95% *CI*	[0.01, 0.45]	[− 1.13, 0.31]	[0.17, 0.80]	[0.33, 1.36]	[0.25, 1.94]
Number of studies	*N* = 6	*N* = 1	*N* = 4	*N* = 2	*N* = 1

The meta-regression performed using the timing of T1 as moderator (*N* = 14) showed that the test on the moderator was not significant, *χ*^2^(1) = 1.40, *p* = 0.23, *estimate* = 0.09. The heterogeneity remained significant, *Q*_*T*_ = 21.30, *p* = 0.04, and Higgins’ *I*^2^ remained stable, *I*^2^ = 43%. The lack of effect of the moderator suggests that timing of T1 does not play a role in determining the differences in the effects reported by the various studies.

## Discussion

To the best of our knowledge, this is the first meta-analysis of RCTs on the impact of HRVB on the reduction of depressive symptoms in different pathological conditions in adult samples.

We selected and analysed 14 published RCTs, including a total of 794 subjects, and examined the effectiveness of HRVB for symptoms of depression in adults compared to control conditions or other active treatments.

Overall, we observed that the HRVB exert a positive and statistically significant (moderate) effect in reducing depressive symptoms after intervention, compared to other control and active conditions. This is partially in line with the significant effect found in the recent meta-analysis on HRVB and/or paced breathing by Lehrer et al.^40^. Considering the previous recent meta-analysis, which found a significant yet small effect size of HRV on depression, we found a slightly higher effect. Such a difference might be due to the fact that Lehrer et al.^40^ assessed the efficacy of both HRVB and paced-breathing and that we included five recent studies^43,51–54^, which were not included in the previous meta-analysis.

We found statistically significant heterogeneity, indicating moderate variance across the included studies. However, when testing for the role of two (i.e., the year of publication of the study and the test used to evaluate depressive symptoms) of the three moderators included in the meta-regressions, heterogeneity decreased and became not significant. Conversely, the timing of T1 did not moderate the observed effect, a result which is in line with the previous meta-analysis^40^, which found that the length of interventions did not influence the effect size.

The effects of the moderators “year of publication” and “questionnaire used” were significant, suggesting that both predictors played a role in determining the effect of HRVB on depressive symptoms.

The year of publication moderated the effect of the intervention, in the direction of larger effect sizes for recent studies. The most recent studies we included had usual care and active control groups and conducted the interventions on participants with heterogeneous features (cardiovascular disease, psychiatric illnesses and no medical condition)^43,51–54^, thus it is unlikely that those specific features had an influence in moderating the effects.

It is possible that in recent years biofeedback devices may have become easier and more user-friendly for participants, capable of giving more sophisticated visual feedback, and thus contributing to increased effectiveness of HRVB.

Considering the effect of the test employed, we found that the significant positive *Hedges’ g* ranged from small to high effect sizes (0.23–1.10) and that the only questionnaire which was associated with a non-significant effect was CES-D. These findings should be interpreted with caution, since only one study employed CES-D as an instrument^46^ and it was the unique study that did not find an improvement of depressive symptoms. Thus, the numerosity of the studies using a specific questionnaire might have influenced the significance of the moderation. However, considering these results, we believe that future studies should be conducted in which the specific features of depression of interest to researchers and clinicians are carefully chosen, with particular consideration given to the time range of the interventions.

In the present meta-analysis, the questionnaires were designed to assess the presence, the severity or the frequency of depressive symptoms only (BDI-II, CES-D, PHQ-9) or of depressive signs together with other symptoms (HADS, DASS) in a time period ranging from 1 week prior to the administration (CES-D, HADS, DASS) to 2 weeks before (PHQ-9, BDI-II) among heterogenous samples. These might be among the reasons why questionnaires were found to moderate the efficacy of the interventions. We speculate that for HRVB studies, questionnaires that screen for the presence of symptoms within 1 week before the time of administration might provide a more precise picture of the efficacy of the interventions, since those that measure the presence of symptoms for 2 weeks before have a fair degree of overlap with the period of the intervention itself.

We consider the results of the present meta-analysis to be reliable, due to our test and adjustment for publication bias. Specifically, we utilized the rank-based trim-and-fill method, which assesses and adjusts results for publication bias depending on funnel plot asymmetry. According to the trim-and-fill method and to the Egger’s test, our results were minimally impacted by publication bias.

Furthermore, the robustness of results, evaluated through a sensitivity analysis, yielded results consistent with the conclusion that HRVB interventions have a positive effect on depressive symptoms. That is, the exclusion of one study at a time through the sensitivity analysis showed the results are not driven by the effect size of only one study. Indeed, the effect size ranged between.33 and 0.42 with a low standard deviation (*SD* = 0.03).

Additionally, consistent with our findings, two studies excluded in the selection procedure due to lack of data^41,42^ reported a decrease in depressive symptoms in biofeedback groups compared to the control group.

### Clinical implications

Depression is one of the most widespread mental diseases, and it occurs in people of all ages across all world regions with more than 264 million people affected (World Health Organization^1^). Furthermore, people with multimorbidity are two to three times more likely to have depression compared to people without multimorbidity or those who have no chronic physical condition^66^.

Autonomic changes are often found in altered mood states and appear to be a central biological substrate linking depression to several physical dysfunctions^23^.

Among autonomic indexes, heart rate variability (HRV) is a significant health marker. Critically, the decrease in HRV that occurs during depression states does not return to normal levels as a consequence of existing psychotherapy or pharmacological treatment, even when the psychological outcome is positive^26^. It is worth noting that HRV may also inform research into the prevention and treatment of depression in later life^24^.

Our findings suggest that HRVB is an effective intervention for the reduction of depressive symptomatology when compared to control or active conditions and, even more importantly, HRVB yielded an effect size that is comparable to other broadly applied approaches (such as CBT)^67^. Interestingly, HRVB intervention is effective also in the treatment of anxiety and perceived stress, with a high reduction of symptoms (*Hedges’ g* = 0.83) in treated groups compared to controls^39^. As a consequence, HRVB might constitute a valuable intervention for patients with symptoms of both anxiety and depression, which often co-occur in the same individual and that can be considered bi-directional risk factor for one another^68^.

Furthermore, the possibility of treating depressive symptoms (and anxiety) among patients with other physical diseases, might render HRVB a suitable intervention for patients with both distressing physical conditions and an emotional burden, such as cancer patients^69,70^.

### Limitations and future directions

To date, there is no specific evidence on which specific pathophysiological conditions, among those included in the present meta-analysis, might derive most benefit from HRVB intervention; nor can it be concluded which biofeedback protocol and devices yield the best results.

As things currently stand, conclusions on specific subsamples or on the severity of the symptoms cannot be drawn. More RCTs are warranted to clarify the effect on specific samples and to perform subgroup analyses according to clinical characteristics of the sample. Such analyses would lead to the possibility of personalizing the interventions based upon the particular characteristics of each individual patient^71,72^.

Furthermore, the measurements of depression in patients presently rely on a subjective scale, and even though we included studies which made use of reliable and standardized scales, the lack of objective assessment might have introduced a risk of bias in the measurement of the relevant outcomes. Thus, measurement tools that provide data with higher reliability and validity should be utilized in future studies, possibly employing objective measurements, such as neuroimaging data^73^. Furthermore, questionnaires might be used to assess which specific depressive features are alleviated by biofeedback (for example, somatic complaints and cognitive signs).

## Conclusions

According to the present meta-analysis, HRVB offers a useful tool for treating depressive symptoms in patients with psychological or medical diseases, although its effectiveness on specific conditions remains unclear. Further studies are warranted to assess which specific HRVB protocols lead to greater results for treating depressive symptoms among adults.

## Supplementary Information

Supplementary Information 1.

Supplementary Information 2.
