# Supplementary material for: A meta-analysis on heart rate variability biofeedback and depressive symptoms
Source: Sci Rep. 2021 Mar 23;11:6650. doi: 10.1038/s41598-021-86149-7 (PMC7988005; doi:10.1038/s41598-021-86149-7)
Supplement: Supplementary file 1 — Supplementary Information 1. [file 41598_2021_86149_MOESM1_ESM.docx]

**Title: A meta-analysis on Heart Rate Variability Biofeedback and depressive symptoms**

Silvia F.M. Pizzoli^1,2*^, MSc, Chiara Marzorati^2^, PhD, Daniele Gatti^3^, MSc, Dario Monzani^1,2^, PhD, Ketti Mazzocco^1,2^, PhD, Gabriella Pravettoni^1,2^, PhD.

^1^ Department of Oncology and Hemato-Oncology, University of Milan, Milano; Italy

^2^ Applied Research Division for Cognitive and Psychological Science, European Institute of Oncology IEO, IRCCS, Milano; Italy

^3^ Department of Brain and Behavioral Sciences, University of Pavia, Pavia. Italy.

*Corresponding author

*Appendix A - Search Strategy*

| **SEARCH STRATEGY FOR SCIENTIFIC LITERATURE** | | | | |
| --- | --- | --- | --- | --- |
| **Search Engine:** | **Search String:** | **Hits** | **Relevant^a^** | **Included^b^** |
| Pubmed | ("heart rate variability biofeedback" OR "HRV biofeedback") AND ("depression" OR "depressive") | 46 | 23 | 12 |
| Embase | ('heart rate variability biofeedback' OR 'hrv biofeedback') AND ('depression' OR 'depressive') | 67 | 38 | 13 |
| PsycINFO | (("heart rate variability biofeedback" or "HRV biofeedback") and ("depression" or "depressive")).af. | 487 | 63 | 14 |
| ProQuest | ("heart rate variability biofeedback" OR "HRV biofeedback") AND ("depressive" OR "depression") | 121 | 10 | 0 |
| **Subtotal** |  | 721 | 134 | 39 |
| **Duplicates** |  |  |  | 23 |
| **Excluded due to missing data** |  |  |  | 2 |
| **Total** |  | 721 | 134 | 14 |
| ^a^ Relevant: number of relevant articles based on title, abstract, and keywords | | | | |
| ^b^ Included: number of included articles based on full article | | | | |
