# Supplementary material for: A meta-analysis on heart rate variability biofeedback and depressive symptoms
Source: Sci Rep. 2021 Mar 23;11:6650. doi: 10.1038/s41598-021-86149-7 (PMC7988005; doi:10.1038/s41598-021-86149-7)
Supplement: Supplementary file 2 — Supplementary Information 2. [file 41598_2021_86149_MOESM2_ESM.docx]

**Title: A meta-analysis on Heart Rate Variability Biofeedback and depressive symptoms**

Silvia F.M. Pizzoli^1,2*^, MSc, Chiara Marzorati^2^, PhD, Daniele Gatti^3^, MSc, Dario Monzani^1,2^, PhD, Ketti Mazzocco^1,2^, PhD, Gabriella Pravettoni^1,2^, PhD.

^1^ Department of Oncology and Hemato-Oncology, University of Milan, Milano; Italy

^2^ Applied Research Division for Cognitive and Psychological Science, European Institute of Oncology IEO, IRCCS, Milano; Italy

^3^ Department of Brain and Behavioral Sciences, University of Pavia, Pavia. Italy.

*Corresponding author

*Appendix B: Characteristics and effect sizes of the included studies*

| **Author** | **Country** | **Type Of Disease** | **Age M (SD)** | **Sample Size (I/C)** | **Gender (M/F)** | **Experimental Group** | **Control Group** | **Physiological Outcome Indicators** | **Psychological Outcome Indicators** | **Scale/Questionnaires** | **Intervention Duration** | **Effect size (*g*)** | **var** |
| --- | --- | --- | --- | --- | --- | --- | --- | --- | --- | --- | --- | --- | --- |
| Amjadian et al.  (2020) | Iran | Patients with coronary artery bypass graft | I:56 (9.51)  C: 59.55 (7.2) | 20/20 | I: 15/5  C: 14/6 | 2-h weekly sessions for 8 weeks | Usual care | HRV | Stress, anxiety, depression | DASS-21 | T0: pre-intervention  T1: post-intervention | 1.03 | 0.11 |
| Brinkmann et al.  (2020) | Germany | Healthy participants with work-related stress | I:42.06 (11.96)  C:42.89 (10.71) | 18/19 | I:6/12  C:5/14 | HRVB training:  30 min daily for 6 weeks | Waitlist:  combined training between mindfulness and biofeedback | HRV (SDNN, RMSSD); cortisol | Chronic stress, stress coping, coping strategies, depression, psychological wellbeing, mindfulness, self-compassion | TICS, SVF, BDI-II, HEALTH-49, FFA-14, SCS | T0: pre-intervention  T1: post-intervention  T2:12 weeks after T1 | 0.3 | 0.10 |
| Burch et al.  (2020) | USA | Cancer survivors | I:60 (3)  C:59 (2) | 17/17 | I:5/12;  C:0/17 | HRVB training:  weekly training sessions for 4/6 weeks + 15 min daily home practice | Usual care | HRV (SDNN, RMSSD, HF, LF) | Pain, stress, distress, fatigue, depression, PTSD, sleep | BPI, PSS, SDI, MFI, BDI-II,  PCL-C, ISQ | T0: pre-intervention  T1: HRVB training completed | 0.34 | 0.11 |
| Chang et al. (2020) | Taiwan | Cardiovascular disease -Ischemic stroke | I:67.6 (11.4);  C: 67.2 (7.6) | 19/16 | I:10/9;  C:8/8 | HRVB  4 BF training sessions over 4 days + 5 phone calls | Usual care | HRV (LF; HF; TP) | Cognitive impairment,  Depression, anxiety | MMSE, HADS | T0: pre-intervention  T1: 1 month after T0  T2: 3 months after T0 | 1.04 | 0.13 |
| Tatschl et al.  (2020) | Austria | Psychiatric inpatients with depressive symptoms | I: 49.03 (7.7)  C: 49.43 (10.4) | 34/35 | I: 12/22  C: 14/21 | HRVB training:  10-min HRVB twice a day for 5 weeks | Usual care | HRV (SDNN, RMSSD, HF, LF) | Depression | BDI-II | T0: pre-intervention  T1: 5 weeks after T0  T2: 1-year after T0 | 0.31 | 0.06 |
| Van der Zwan et al. (2019) | Netherlands | Healthy pregnant or not-pregnant women with stress | I: 31.8 (5.9);  C: 31.3 (6.1) | 29/21 | All females | HRVB training: 5-week meetings of 60 to 90 min duration each. | Waitlist: HRVB between T1 and T2) | HRV | Depression, anxiety, sleep quality, well-being | DASS, PSQI, SPW | T0: pre-treatment  T1: HRVB training completed  T2: 6 weeks later T1 | 0.86 | 0.09 |
| Caldwell et al. (2018) | USA | College students with MDD | I: 20.09 (1.81)  C: 20.20 (1.47) | 10/10 | All females | HRVB: 4-5 weekly sessions for 5 weeks  + psychotherapy | Psychotherapy | HRV (SDNN, HF, LF, HF/LF) | Depression | BDI-II | T0: pre-intervention  T1: post-intervention  T2: follow-up 6 weeks after T0 | 0.01 | 0.18 |
| Yu et al. (2018) | Taiwan | Patients with Coronary Artery Disease | I: 61.24 (7.42);  C: 60.31 (6.87) | 75/59 | I:66/9  C:53/6 | 6 sessions of HRVB: weekly 60-min session | Usual care and 10 min of  psychological education after post-intervention assessment | Breathing rate,  blood pressure,  HRV (LF; HF; TP; LF/HF ratio) | Depression, hostility traits | BDI-II, CHI-SF | T0: pre-intervention  T1: post-intervention  T2: 1-year follow-up after intervention | 0.25 | 0.03 |
| Windthorst et al. (2017) | Germany | Patients with Chronic fatigue syndrome | I: 51.4 (8.1);  C: 50.0 (10.9) | 13/11 | All females | HRVB: 8 individual training sessions of 50 min each at weekly intervals | Graded exercise training | HRV | Fatigue, general health perception, quality of life, depression | MFI, SF36, PHQ-9 | T0: pre-intervention  T1: post-intervention  T2: follow up 5-month after | 1.1 | 0.18 |
| Penzlin et al. (2015) | Germany | Patients in rehabilitation treatment for alcohol use disorder | I: 40 (±7)  C: 44 (±8) | 24/24 | I:17/7  C:17/7 | 3 20-minutes sessions of HRVB training per week over 2 weeks  + usual care | Usual care | HRV, vasomotor function | Craving, anxiety, depression | Obsessive Compulsive Drinking Scale, SCL-90, BDI-II | T0: pre-intervention  T1: post-intervention  T2: 3 weeks after T1  T3: 6 weeks after T1 | 0.08 | 0.08 |
| Van der Zwan et al. (2015) | Netherlands | University students aged between 18 and 40 years old | I:26.99 (6.53);  C:25.28 (4.42) | 25/23 | I:8/17  C:5/18 | 5-week sessions of HRVB; daily exercises at home increasing in duration over time | 5-week sessions of physical daily exercises at home increasing in duration over time | HRV | Depression, anxiety, stress, sleep, well-being | DASS, PSQI, SPW | T0: pre-intervention  T1: post-intervention  T2:6 weeks after T1 | -0.09 | 0.08 |
| Gruzelier et al. (2014) | UK | First year contemporary dance conservatoire students | NA | 16/16 | NA | 10 sessions (twice a week) of HRVB | No treatment | HRV | Creativity, anxiety, depression, personality | Alternate Uses Test, DASS,  NEO PI-R | T0: pre-intervention  T1: 5 weeks after T0 | 0.24 | 0.13 |
| Hallman et al. (2011) | Sweden | Patients with neck-shoulder pain and stress related symptoms | I: 40 (9)  C:43 (4) | 12/11 | I:1/11  C:1/10 | 10-weeks sessions of HRVB | No treatment | HRV, Hand grip, cold pressor, breathing | Quality of life, pain, stress-related symptoms, anxiety and depression | SF-36 , Borg CR10 Scale , SMSS, HADS | T0: pre-intervention  T1: 10 weeks after T0 | 0.66 | 0.13 |
| Swanson et al. (2009) | USA | Patients with heart failure | I:54 (±11)  C:56.4 (±13.5) | 100/100 | I:80/20  C:79/21 | 6-weeks HRVB training once per week for 45 min | Placebo: quasi-false alpha-theta EEG  biofeedback training | HRV | Heart failure influence, positive and negative affect, depression, expectancy, stress | Minnesota Living with Heart Failure Questionnaire, PANAS, CES-D, Credibility/Expectancy Questionnaire, Stress Management and Exercise Practices Questionnaire  Daily Stress Management and Exercise Record | T0: pre-intervention  T1: 6 weeks after T0  T2: 18 weeks after T0 | -0.41 | 0.13 |

*Legend: I: intervention group; C: control group; HRVB: heart rate variability biofeedback; LF: low frequency; HF: high frequency; TP: total power; DASS-21: Depression Anxiety Stress Scales Short Form; BPI: Brief Pain Inventory; PSS: Perceived Stress Scale; SDI: Suscro Distress Inventory; MFI: Multidimensional Fatigue Inventory; PCL-C: PTSD Check List—Civilian Version; ISQ: Insomnia Symptom Questionnaire; TICS: Trier Inventory for Chronic Stress; SVF: Stressverarbeitungsfragebogen german questionnaire; HEALTH-49: Hamburg Modules for the Assessment of Psychosocial Health in Clinical Practice; FFA-14: Freiburg Mindfulness Inventory; SCS: self-compassion scale; MMSE: Mini-Mental State Examination; HADS: Hospital Anxiety and Depression Scale; DASS: Depression Anxiety Stress Scales; PSQI: Pittsburgh Sleep Quality Index; SPW: Scales of Psychological Well-being; BDI-II: Beck Depression Inventory II; CHI-SF: Chinese Hostility Inventory-short form; MDD: major depressive disorder; SF36: Short Form General Health Survey; MFI: Multidimensional Fatigue Inventory; SCL-90 R: Symptom Checklist-90-Revised; SMSS: stress medicine symptom scale; PANAS: Positive and Negative Affect; CES-D: Center for Epidemiological Studies-Depression Scale..NA: data not reported.*
